# High-standard predictive equations for estimating body composition using bioelectrical impedance analysis: a systematic review

**DOI:** 10.1186/s12967-024-05272-x

**Published:** 2024-05-29

**Authors:** Francesco Campa, Giuseppe Coratella, Giuseppe Cerullo, Zeasseska Noriega, Rubén Francisco, Davide Charrier, Alfredo Irurtia, Henry Lukaski, Analiza Mónica Silva, Antonio Paoli

**Affiliations:** 1 Department of Biomedical Sciences, University of Padua, Padua, Italy; 2 Department of Biomedical Sciences for Health, Università degli Studi di Milano, Milan, Italy; 3 NEFC-Barcelona Sports Sciences Research Group, Institut Nacional d’Educació Física de Catalunya (INEFC), Universitat de Barcelona (UB), 08038 Barcelona, Spain; 4 Exercise and Health Laboratory, CIPER, Faculdade de Motricidade Humana, Universidade de Lisboa, Cruz-Quebrada, Portugal; 5 Department of Kinesiology and Public Health Education, Hyslop Sports Center, University of North Dakota, Grand Forks, USA

**Keywords:** BIA, Fat mass, Fat-free mass, Total body water, Resistance training, Fitness

## Abstract

The appropriate use of predictive equations in estimating body composition through bioelectrical impedance analysis (BIA) depends on the device used and the subject’s age, geographical ancestry, healthy status, physical activity level and sex. However, the presence of many isolated predictive equations in the literature makes the correct choice challenging, since the user may not distinguish its appropriateness. Therefore, the present systematic review aimed to classify each predictive equation in accordance with the independent parameters used. Sixty-four studies published between 1988 and 2023 were identified through a systematic search of international electronic databases. We included studies providing predictive equations derived from criterion methods, such as multi-compartment models for fat, fat-free and lean soft mass, dilution techniques for total-body water and extracellular water, total-body potassium for body cell mass, and magnetic resonance imaging or computerized tomography for skeletal muscle mass. The studies were excluded if non-criterion methods were employed or if the developed predictive equations involved mixed populations without specific codes or variables in the regression model. A total of 106 predictive equations were retrieved; 86 predictive equations were based on foot-to-hand and 20 on segmental technology, with no equations used the hand-to-hand and leg-to-leg. Classifying the subject’s characteristics, 19 were for underaged, 26 for adults, 19 for athletes, 26 for elderly and 16 for individuals with diseases, encompassing both sexes. Practitioners now have an updated list of predictive equations for assessing body composition using BIA. Researchers are encouraged to generate novel predictive equations for scenarios not covered by the current literature.

*Registration code in PROSPERO*: CRD42023467894.

**Supplementary Information:**

The online version contains supplementary material available at 10.1186/s12967-024-05272-x.

## Introduction

The determination of body composition is a common practice for evaluating health and nutritional status, as well as monitoring the effects of training or diet strategies [1, 2]. While a simplistic approach involves assessing body composition based on body mass and its changes over time, breaking it down into different components enables a more meticulous and in-depth evaluation [3, 4]. At the molecular level of body composition analysis [4] initial attempts to break down body mass were based on a two-component model, categorizing it into fat (FM) and fat-free mass (FFM), also known as lean mass [5]. It is worth noting that FFM is often also referred to by synonyms such as lean mass or lean body mass [6]. However, monitoring FFM alone can obscure meaningful changes in body composition since its composition comprises several components (e.g., water, protein, and minerals) not considered in the assessment [5, 6].

To overcome the limitations of the two-component model and enhance the accuracy of body composition analysis, various multi-component models have been developed over time [2]. These models necessitate the use of one or more methods, including dual-energy X-ray absorptiometry (DXA) for bone mineral assessment, dilution techniques for total-body water, hydrostatic weighing or air displacement plethysmography for body volume, total-body potassium for body cell mass, and magnetic resonance imaging (MRI) and computed tomography for skeletal muscle or other tissues and organs [5]. For example, Wang et al. [3] presented a procedure based on a four-component model that is currently considered the state-of-art method for determining FFM and FM. Additionally, the use of multi-components models allows for the breakdown of FFM into different parts such as total body water (TBW), intra (ICW) and extracellular water (ECW), body cell mass (BCM), lean soft mass (LSM), skeletal muscle mass (SMM), and bone mass [7, 8]. With these procedures available to researchers, predicting body mass components by assuming constant hydration of FFM (i.e., TBW/FFM = 0.73) or neglecting the potential variation in FFM and FM density [6] cannot be considered as a valid approach for assessing body composition. Indeed, several dated studies have employed coefficients such as this to derive FFM and subsequently FM based on TBW predictions, or they have relied on density assumptions to derive FFM and FM using only densitometry techniques (i.e., hydrostatic weighing or air displacement plethysmography) [9–11]. In particular, the practice of deriving FFM by assuming that TBW represents a constant fraction of it has led to BIA being incorrectly identified as a hydration-dependent method. Therefore, a comprehensive determination of body mass components using multi-components models enables a valid assessment of body composition in different populations, especially those with non-conventional hydration or density properties (e.g., children, athletes, or older individuals) [6, 12].

Considering the limitations of densitometric, hydrometric, or imaging techniques for quantifying body composition due to high costs, lengthy procedures, and non-user-friendly processes, alternative methods such as bioelectrical impedance analysis (BIA) or surface anthropometry are often employed for routine assessments [13–16]. The theoretical basis of BIA revolves around the conductivity properties of biological tissues, quantified as bioelectrical resistance and reactance, deriving by conductor volumes (i.e., lean soft components) [17]. Bioelectrical resistance represents the opposition offered by the body to the flow of an alternating electrical current and is inversely related to the water and electrolyte content of tissues [17]. Bioelectrical reactance is related to the capacitance properties of the cell membrane and variations that can occur depending on its integrity, function, and composition [17]. Notably, these properties can be attributed to theoretical models, according to which biological tissues are traditionally conceived as electrical circuits arranged in series with each other [18]. Starting from the relationships between resistance and reactance with TBW [19], numerous BIA-based predictive equations have been developed over the years [20]. Previous studies have demonstrated that the use of different BIA technologies (i.e., hand to hand, leg to leg, foot to hand, and segmental) and sampling frequencies results in different outputs, so that these equations cannot be interchangeable between different devices [21–24]. Additionally, the choice of the equation should be made considering the subjects' characteristics, such as chronological and biological age, geographical provenance, sex, health status, and level of physical activity [25–27]. Therefore, the choice of the appropriate predictive equation is crucial to ensure the validity of the body composition estimation. A further question is that various studies including those providing reference data based on BIA do not disclose the procedures used [24, 28]. Many of these studies merely mention the type of software employed, making it impossible to discern which formulas were used to convert raw bioelectrical parameters into components of body mass. Furthermore, such data may no longer be representative over time because companies producing such software can alter the equations without notifying users [29]. While this approach is undoubtedly quicker than a systematic and accurate selection of the most appropriate equation among those available in the literature, does not guarantee a high-standard validity.

**Fig. 1:**
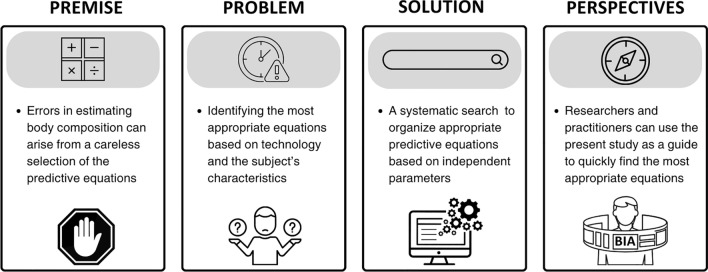
A schematization of the background and objectives set for the current study

The literature is now replete with predictive equations developed for estimating body composition using BIA. However, several limitations may question the validity of some equations. First, the use of the two-component model instead of a multi-component model approach. Second, predicting FFM and FM starting from hydration or density assumptions. Third, the development of predictive models without using criterion methods. Fourth, mixing different populations without including specific factors (e.g., age, maturity status, sex, physical activity level) as independent variables in the predictive models [30]. Therefore, the present review aimed to examine the relevant literature to extract all the predictive equations currently available, and list only those free of the aforementioned limitations. In addition, we provided a clear organization of the predictive equations based on the BIA technology, sampling frequency, and population characteristics. Such a list will help scientists and practitioners select the most appropriate predictive equations in accordance with the BIA devices and population peculiarities. Summarizing the state-of-the-art of BIA-based prediction of body composition will also be helpful to optimize the development of new predictive equations considering what is currently lacking in the literature. Obtaining accurate results would also allow for utilizing the data to evaluate other health-related parameters through estimating basal metabolism or the quantity of macronutrients based on body composition data [31]. Figure 1 depicts the premise and the problem at the basis of the present study, presenting the aims and future perspectives.

## Methods

### Search strategy and eligibility criteria

The present study was carried out following the Preferred Reporting Items for Systematic reviews and Meta-Analyses (PRISMA) guidelines [32]. The two independent researchers (F.C. and Z.N.) conducted systematic searches in Scopus, and PubMed on December 22th, 2023, identifying potential eligible studies without any restriction related to year of publication. A search query included combinations of at least one of the terms identifying BIA, with at least one of the terms regarding the study design, a term regarding reference techniques, and a term referring to body composition. We composed different query strings depending on the variability of the databases functioning, using the following terms: (bioelectrical impedance analysis) OR (bioimpedance) OR (BIA) AND (development) OR (new predictive equation) OR (prediction) OR (estimation) OR (Dual Energy X-ray absorptiometry) OR (dilution techniques) OR (deuterium dilution) OR (tritium dilution) OR (bromide dilution) OR (magnetic resonance imaging) OR (computerized tomography) OR (total-body potassium) OR (DXA) OR (MRI) AND (fat mass) OR (fat-free mass) OR (total body water) OR (body fluids) OR (extracellular water) OR (intracellular water) OR (body cell mass) OR (skeletal muscle mass) OR (lean soft mass) OR (lean soft tissue) OR (FM) OR (FFM) OR (TBW) OR (ECW) OR (ICW) OR (BCM) OR (SMM) OR (LSM) OR (LST) AND (three-compartment model) OR (four-compartment model) OR (3C model) OR (4C model).

The inclusion criteria were as follows:

- Peer-reviewed articles that developed at least one predictive equation for estimating body mass components (i.e., FM, FFM, TBW, BCM, LSM, and SMM) using BIA.
- Accessible in English in full text.

The exclusion criteria were as follows:

- Research protocols, theses/dissertations, abstracts, letters to the editor, case reports, book chapters, guidelines, position papers, and unpublished works.
- Articles aimed at developing predictive equations without recognized reference techniques or considering a body composition assessment based on a two-component model for FM and FFM.
- Articles aimed at validating predictive equations.
- Articles where the characteristics of the BIA devices (e.g., technology and sampling frequency) were not available.
- Articles where predictive equations were developed by mixing different populations without including variables such as sex, maturity status, or age in the development of regression models. For example, if an equation included participants of both sexes and a sex code was included among the independent variables, the equation can be considered eligible. On the contrary, an equation including participants of both sexes without including a sex code in the predictive model cannot be considered eligible. Similarly, an equation including participants aged from 15 to 90 years old that does not require the age as independent variable was excluded from the review. Moreover, studies developing predictive equations where participants of different health status were mixed were not considered eligible.

### Study selection and data processing

Based on the initial titles retrieved, duplicates were removed. After concluding the search, all records were compiled into the Endnote for Windows version X9, 2018 (Clarivate, Philadelphia, USA) software to delete all duplicates showing the same: (a) title, authors and year of publication and (b) title, authors, and journal title. The records remaining after the deletion of duplicates were exported to an Excel file for Windows version 16.75.2 (Microsoft, Washinton, EUA) organized based on essential information for screening, such as authors’ names, publication year, journal title, digital object identification (DOI), article title and abstract. Abstracts identified from the literature searches were screened for potential inclusion by two authors (F.C. and Z.N.) and a third author (G.C.) when there was a disagreement between the first two. Data extraction included information about each article, such as: authors, year, reference methods, participants’ information (gender, age, sports, diseases, geographical ancestry), bioelectrical impedance techniques and devices, predictive equations, and their characteristics. The selected studies were grouped in five categories, such as under 18 years old subjects, adults, athletes, elderly, and people with disease.

### Quality assessment

Two reviewers independently assessed the quality of the included studies with the Quality Assessment of Diagnostic Accuracy Studies (Quadas-2) [33]. This scale has two dimensions (risk of bias and applicability concerns) and four domains (patient selection, index test, reference standard and flow and timing) that are scored with unclear risk, low risk, or high risk. Differences were resolved through discussions and consultations with a third researcher.

## Results

### Study selection

**Fig. 2:**
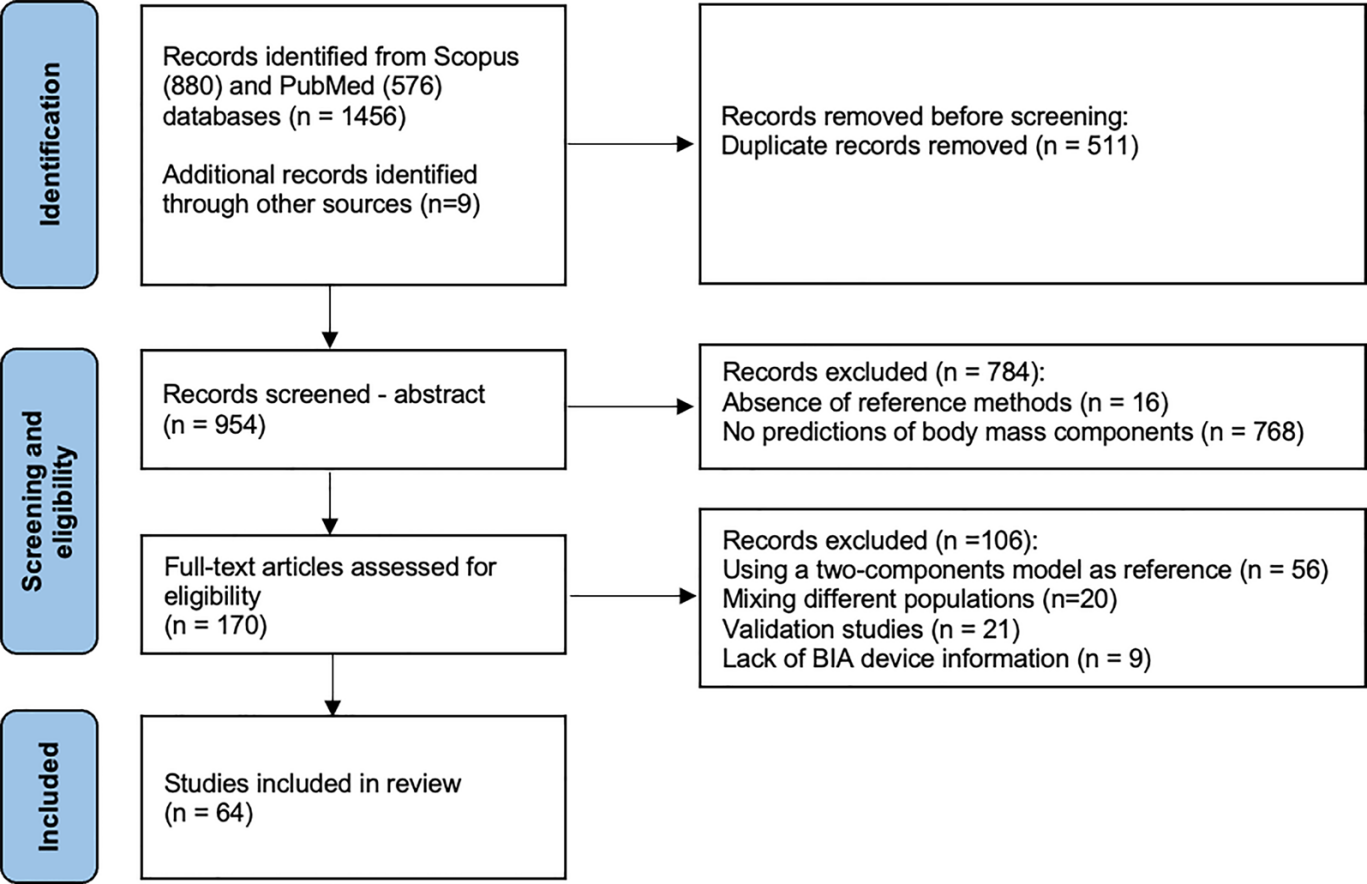
PRISMA Flow chart of the studies’ selection

A total of 106 BIA-based predictive equations resulted from the 64 studies included in the review. The PRISMA flow chart is shown in Fig. 2.

### Risk of bias

A detailed view of the quality assessment is presented in Supplementary Table 1. Overall, the quality of studies was good. Risk of bias was high in two [34, 35] and unclear in six [36–41] studies due to the low to moderate (R^2^ < 80%) coefficient of determination (R^2^) resulted from the multiple regression models. Concerns with applicability were high in 19 studies [37, 42–59] and unclear [41, 50, 60–69] in 12 studies due to the restricted sample size.

### Participants

**Fig. 3:**
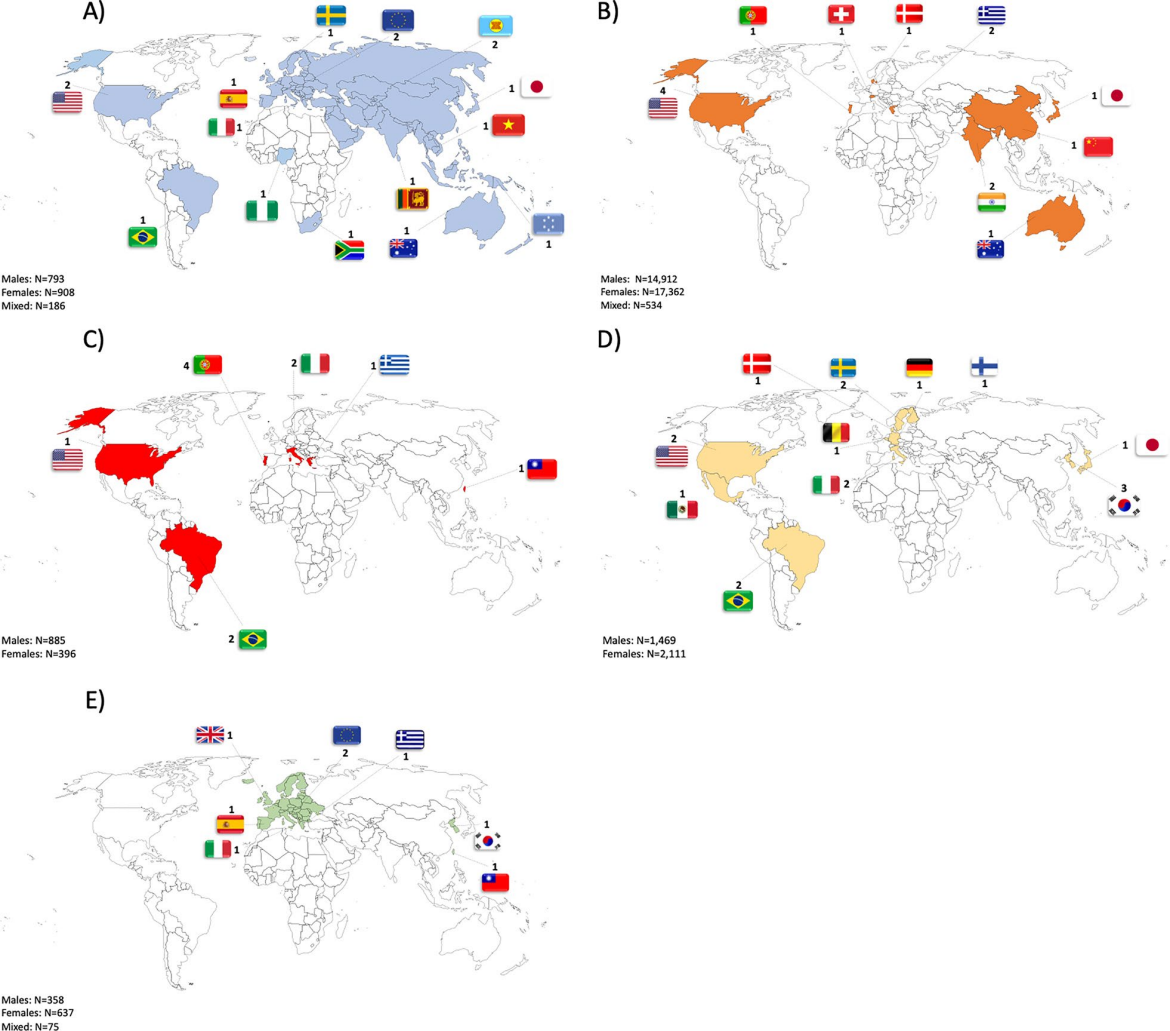
World map with number of included studies for under 18 years old subjects (**A**), adults (**B**), athletes (**C**), elderly (**D**), and people with disease (**E**)

A total of 40,626 individuals were involved in the present systematic review. Out of the total number, 18,417 participants were males and 21,414 females. Three studies involving 186 [70], 72 [52], 462 [71], and 75 [65] subjects did not specify the sex of the participants. A total of 793 males, 908 females, and 186 subjects without sex info were included in 14 studies [37, 43–45, 47, 55, 57–59, 72–76] aimed to develop predictive equations for subjects aged < 18 years. A total of 14,912 males, 17,362 females, and 534 subjects without any sex designated were included in 14 studies [34, 42, 47, 50, 52, 54, 61, 66, 71, 77–82] aimed to develop predictive equations for adults (from 18 to 65 years old) from the general population. A total of 885 males and 396 females were included in 11 studies [31, 39, 48, 51, 62, 69, 83–88] aimed to develop predictive equations for athletes. A total of 1469 males and N = 2111 females were included in 17 studies [36, 38, 46, 53, 60, 63, 67, 68, 89–97] aimed to develop predictive equations for elderly people. A total of 358 males, 637 females, and 75 subjects without sex designated were included in 8 studies [35, 40, 41, 56, 64, 65, 98, 99] aimed to develop predictive equations for people with diseases. The number of the studies for each geographical area us depicted in Fig. 2 for each of the considered populations (Fig. 3).

### Bioelectrical impedance-based predictive models according to gender, populations, and technologies

**Fig. 4:**
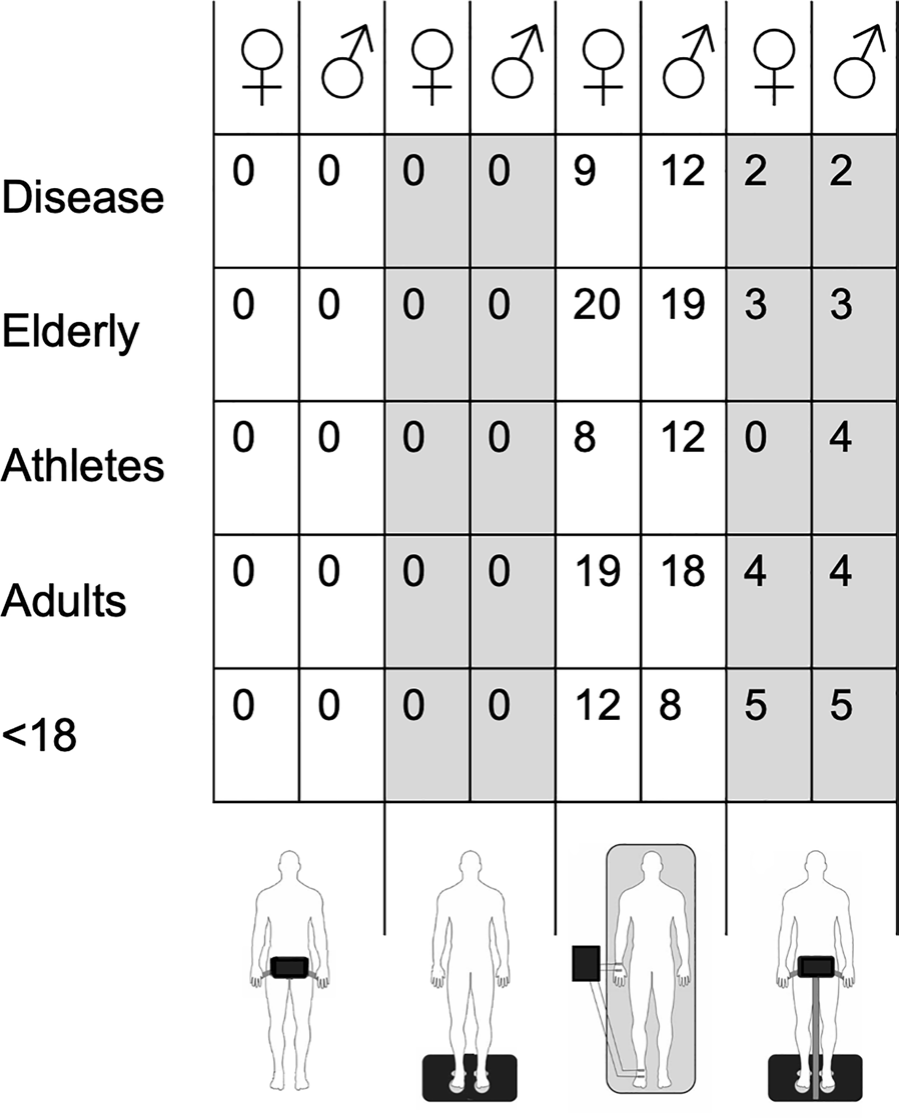
Number of predictive equations available for each BIA technology (e.g., hand to hand, leg to leg, foot to hand, and segmental) according to sex and population

Figure 4 schematizes the number of equations available for each BIA technology (e.g., hand to hand, leg to leg, foot to hand, and segmental) according to sex and population.

**Fig. 5:**
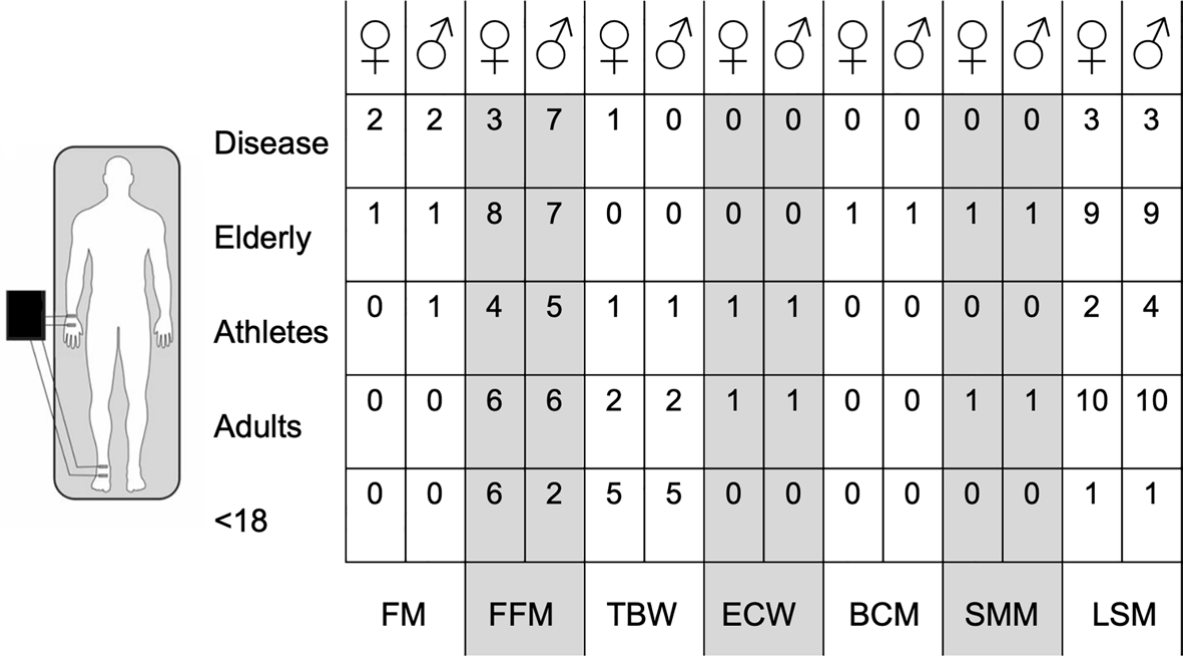
Number of the predictive equations for each estimable body mass component, according to sex and population for the foot to hand technology. *FM* fat mass, *FFM* fat-free mass, *TBW* total body water, *ECW* extracellular water, *ECW* extracellular water, *BCM* body cell mass, *LST* lean soft mass, *SMM* skeletal muscle mass

**Fig. 6:**
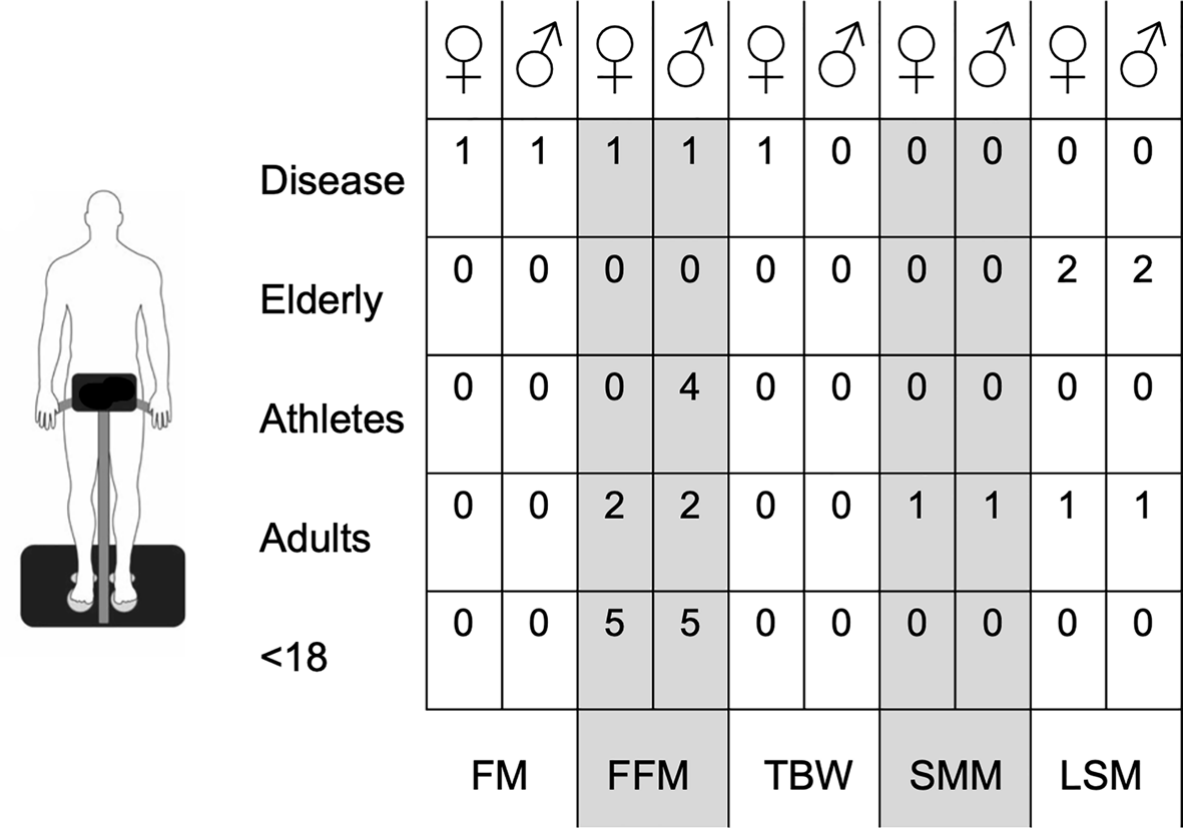
Number of the predictive equations for each estimable body mass component, according to sex and population for the segmental technology. *FM* fat mass, *FFM* fat-free mass, *TBW* total body water, *LST* lean soft mass, *SMM* skeletal muscle mass

The number of the predictive equations for each estimable body mass component, according to sex and population are shown in Figs. 5 and 6, for the foot to hand and segmental technologies, respectively.

### Bioelectrical impedance-based equations for under 18 years old people

Table 1 reports the characteristics of the 19 predictive equations developed in 14 studies on subjects < 18 years old from the general populations. Fifteen predictive equations from 11 studies were developed with a SF-BIA, while four predictive equations from three studies were developed with MF-BIA using bioelectrical parameters measured at 50 kHz.

**Table 1:** Predictive equations (N = 19) from studies (N = 14) on under 18 years people from the general population

	Author	Variable	Analyzer	Reference	Equation	Country and participants characteristics	R^2^	SEE	Note
SF-BIA	*Foot to hand*								
	Guo et al. (1993)	FFM	BIA 101, RJL Systems Inc., USA	DXA	FFM_black girls_ (kg) = – 12.37 + 0.58 × H^2^/R + R + 0.59 × thigh circumference – 0.45 subscapular skinfold + 0.25 suprailiac skinfold FFM_white girls_ (kg) = 3.71 + 0.38 × H^2^/R – 0.57 × arm circumference + 0.54 × Wt + 58.07 × Xc/R	USA, Black girls (N = 31; 11.9 ± 2.1 years) USA, White girls (N = 38; 11.9 ± 2.1 years)	0.97 0.99	1.97 kg 1.14 kg	Height in meters, skinfolds and circumferences are in cm
	Morrison et al. (2001)	FFM	BIA 101, RJL Systems Inc., USA	DXA	FFM_black girls_ (kg) =– 8.78 + 0.78 × H/R + 0.10 × Xc + 0.18 × Wt FFM_white girls_ (kg) = 1.07 + 0.37 × H/R – 0.17 × tricep skinfold + 0.47 × Wt	USA, Black girls (N = 61; 11.2 ± 2.7 years) USA, White girls (N = 65; 11.2 ± 2.4 years)	0.99 0,99	1.95 kg 1.14 kg	Skinfolds and circumferences are in cm
	Leman et al. (2003)	TBW	BIA 101Q, RJL Systems Inc., USA	Dilution techniques	TBW (L) = 1.67 + 0.35 × H^2^/R + 0.24 × Wt – 0.74 × sex	Nigeria, boys (N = 7; 14.4 ± 2.7 years) and girls (N = 22; 11.9 ± 3.9 years)	0.70	0.99 l	Sex code: male = 1, female = 0
	Masuda et al. (2004)	TBW	TP-95 K, Toyo Physical Inc., Japan	Dilution techniques	TBW (kg) = 0.149 × H^2^/R + 0.244 × Wt + 0.460 × age + 0.501 × sex + 1.628	Japan, boys (N = 26; 4.9 ± 0.9 years) and girls (N = 20; 5.2 ± 0.8 years)	0.96	0.44 kg	Sex code: male = 1, female = 0
	Nielsen et al. (2007)	FFM LSM	BIA 103, RJL Systems Inc., USA	DXA	FFM (kg) = – 5.11 × [± 2.19] + H^2^/R × 0.54 × [± 0.05] + Xc × 0.05 [± 0.02] + H × 0.06 [± 0.02] + Wt × 0.09 [± 0.03] + sex × 0.97 × [± 0.20] LSM (kg) = – 3.97 × [± 2.11] + H^2^/R × 0.52 × [± 0.05] + Xc × 0.04 [± 0.02] + H × 0.06 [± 0.02] + Wt × 0.08 [± 0.03] + sex × 0.93 × [± 0.19]	Sweden, boys (N = 52; 9–11 years) and girls (N = 49; 9–11 years)	0.95 0.94	0.87 kg 0.84 kg	Sex code: male = 1, female = 0
	Wickramasinghe et al. (2007)	TBW	Bodystat Ltd, British Isles	Dilution techniques	TBW (L) = 0.41 × H^2^/Z + 0.17 × Wt + 1.1 × sex + 0.44	Sri Lanka, boys (N = 105; 9.5 ± 2.7 years) and girls (N = 83; 10.1 ± 2.8 years)	0.86	2.10 l	Sex code: male = 1, female = 0
	Liu et al. (2011)	TBW	DF50, ImpediMed Ltd, USA	Dilution techniques	TBW (L) = 0.231 × H^2^/R + 0.066 × H + 0.188 × Wt + 0.128 × age + 0.5 × sex – 0.316 × ethnicity – 4.574	Asia, boys (N = 133; 8–10 years) and girls (N = 98; 8–10 years)	0.88	1.3 kg	Sex code: male = 1, female = 0 Ethnicity code: Thai = 1, others = 0
	Kourkoumelis et al. (2021)	TBW	DF50, ImpediMed Ltd, USA	Dilution techniques	TBW (kg) = 0.44 × H^2^/R + 0.12 × Wt + 0.33 × sex + 1.5	Europe, boys and girls (N = 186; 120.8 ± 15.2 months)	0.87	1.81 kg	Sex code: male = 1, female = 0
	Da Costa et al. (2022)	FFM	BIA1010, American Medical do Brasil Ltda, Brazil	DXA	FFM (kg) = – 17.189 + 0.498 × H^2^/R + 0.226 × Wt + 0.071 × Xc – 2.378 × sex + 0.097 × H + 0.222 × age	Brazil, boys (N = 86; 10–18 years) and girls (N = 86; 10–18 years)	0.92	2.49 kg	Sex code: male = 0, female = 1
MF-BIA	*Segmental*								
	Sluyter et al. (2010)	FFM	BC-418, Tanita Corporation, Japan	DXA	FFM_males_ (kg) = 0.607 × H^2^/Z + 1.542 × age + 0.220 × H + 0.096 × Wt + 1.836 × ethnicity – 47.547 FFM_females_ (kg) = 0.531 × H^2^/Z + 0.182 × H + 0.096 × Wt + 1.562 × ethnicity – 15.782	Europe, Asia and Oceania, boys (N = 215; 15.9 ± 1.6 years) and girls (N = 216; 15.9 ± 1.6 years)	0.93 0.91	3.09 kg 2.19 kg	Ethnicity code: European or Asian = 0, Maori or Pacific = 1 Ethnicity code: non-Pacific = 0, Pacific = 1
	Gutiérrez-Marin et al. (2021)	FFM	BC-418, Tanita Corporation, Japan	4C	FFM (kg) = – 9.012 + 0.818 × H^2^/Z + 0.742 × age + 0.648 × sex + 0.235 × BMI	Spain, boys (N = 35; 8–14 years) and girls (N = 31; 8–14 years)	0.88	2.70 kg	Sex code: male = 1, female = 2
	*Segmental*								
	Pietrobelli et al. (2003)	FFM	BIA Human-Im, DS Medigroup, Italy	DXA	FFM_males_ (kg) = 0.6375 × H^2^/Z + 5.9913 FFM_females_ (kg) = 0.7597 × H^2^/Z + 3.5853	Italy, boys (N = 50; 9.7 ± 1.3 years) and girls (N = 25; 10.2 ± 1.6 years)	0.89 0.90	5.12%	Data measured at 50 kHz
	Van Zyl et al. (2019)	FFM	mBCA 514, Seca Corporation, USA	DXA	FFM (kg) = 105.20 + 0.807 × sex + 0.174 × Wt + 0.01 × Xc + 15.71 × logH^2^/R	South Africa, boys (N = 40; 8.6 ± 1.5 years) and girls (N = 44; 8.5 ± 1.4 years)	0.95	0.97 kg	Data measured at 50 kHz Sex code: male = 1, female = 0
	Nguyen et al. (2020)	FFM	mBCA 525, Seca Corporation, USA	DXA	FFM (kg) = 0.299 × H^2^/R + 0.086 × H + 0.245 × Wt + 0.260 × age + 0.901 × sex – 0.415 × ethnicity – 6.952	Vietnam, boys (N = 44; 4–7 years) and girls (N = 39; 4–7 years)	0.91	2.63 kg	Age in months Data measured at 50 kHz Ethnicity code: Vietnamese = 1, others = 0 Sex code: male = 0, female = 1

*BIS* bioelectrical impedance spectroscopy, *DXA* dual-energy X-ray absorptiometry, *FFM* fat-free mass, *H* height (cm), *LSM* lean soft mass, *MF-BIA* multifrequency bioelectrical impedance analysis, *R* resistance, *R*^*2*^ coefficient of determination, *SEE* standard error of estimation, *SF-BIA* single-frequency bioelectrical impedance analysis, *TBW* total body water, *Wt* body mass, *Xc* reactance, *Z* impedance, *4C* 4 compartmental model

### Bioelectrical impedance-based equations for adults from the general population

Table 2 reports the characteristics of the 26 predictive equations developed in 14 studies on subjects from the general populations. Twenty-two predictive equations from 12 studies were developed with a SF-BIA, while four predictive equations from two studies were developed with MF-BIA using bioelectrical parameters measured at 5, 50, and 250 kHz. Only one study provided a predictive equation using BIS at a frequency of 50 kHz.

**Table 2:** Predictive equations (N = 26) from studies (N = 14) on the general adult population

	Author	Variable	Analyzer	Reference	Equation	Country and participants characteristics	R^2^	SEE	Note
SF-BIA	*Foot to hand*								
	Lukaski et al. (1988)	TBW	BIA 101B, RJL Systems Inc., USA	Dilution techniques	TBW (L) = 0.377 × H^2^/R + 0.14 × Wt −–0.08 × age + 2.9 × sex + 4.65	USA, men (N = 25; 29.4 ± 2.4 years) and women (N = 28; 45.1 ± 2.6 years)	0.97	1.50 l	Sex code: male = 1, female = 0
	Heitman et al. (1990)	FFM	BIA 101, RJL Systems Inc., USA	4C	FFM_males_ (kg) = 0.279 × H^2^/R + 0.245 × Wt + 0.231 × H –0.077 × age –14.94 FFM_females_ (kg) = 0.279 × H^2^/R + 0.181 × Wt + 0.231 × H – 0.077 × age –14.94	Danmark, men and women (N = 72; 35–65 years)	0.89	3.30 l	
	Zillikens et al. (1991)	TBW	BIA 101A, RJL Systems Inc., USA	Dilution techniques	TBW (L) = – 0.442 + 0.484 H^2^/R + 0.079 × Xc –0.054 × age + 0.128 × Wt –1.493 × sex + 0.819 × ethnicity	USA, men (N = 83; 19 –50 years) and women (N = 84; 19–50 years)	0.97	1.49 l	Sex code: male = 0, female = 1 Ethnicity code: Caucasian = 0, African American = 1
	Jakicic et al. (1998)	FFM	BIA 101A, RJL Systems Inc., USA	DXA	FFM (kg) = 2.68 + 0.20 × H^2^/R + 0.19 Wt + 2.55 × ethnicity + 0.1157 × H	USA, women (N = 123; 25–45 years)	0.65	8.80 kg	Ethnicity code: Caucasian = 0, African American = 1
	Janssen et al. (2000)	SMM	BIA 101B, RJL Systems Inc., USA	MRI	SMM (kg) = H^2^/R × 0.401 + sex × 3.825 + age × -0.071 + 5.102	USA, multiethnic men (N = 230; 41.9 ± 14.5 years) and women (N = 158; 41.9 ± 14.5 years)	0.86	2.70 kg	Sex code: male = 1, female = 0
	Kyle et al. (2003)	ALSM	Xitron 4000B, Xitron technologies, Inc, USA	DXA	ALSM (kg) = –4.211 + 0.267 × H^2^/R + 0.095 × Wt + 1.909 × sex –0.012 × age + 0.058 × Xc	Switzerland, men (N = 213; 22–94 years) and women (N = 113; 22–94 years)	0.98	1.12 kg	Sex code: male = 1, female = 0
	Kontogianni et al. (2005)	FFM	BIA 101Q, RJL Systems Inc., USA	DXA	FFM (kg) = 17.825 + 0.38 × H^2^/R + 0.172 × Wt –0.156 × age	Greece, women (N = 30; 53.9 ± 6.4 years)	0.88	1.89 kg	
	Rush et al. (2006)	FFM	BIM4, ImpediMed Ltd, Australia	DXA	FFM_males_ (kg) = 0.382 × H^2^/R + 0.167 × Wt + 0.320 × H –36.382 FFM_females_ (kg) = 0.456 × H^2^/R + 0.127 × Wt + 0.0746 × Xc + 5.959	India, men (N = 110; 19–74 years) and women (N = 101; 19–74 years)	0.84 0.70	2.79 kg 2.01 kg	
	Dasgupta et al. (2019)	FFM	Bodystat 1500, Bodystat Ltd, British Isles	DXA	FFM (kg) = 32.637 –0.222 × age –32.51 × WHR + 0.33 × BMI + 1.58 × BWC + 0.510 × waist circumference	India, men (N = 117; 19.6 ± 0.8 years)	N/R	N/R	Height in meters BWC: birth weight code normal = 1, low = 2
	Kanellakis et al. (2020)	FFM	BIA 101, Akern s.r.l., Italy	DXA	FFM (kg) = 12.299 + 0.164 × Wt + 7.287 × sex –0.116 × R/H + 0.365 × Xc/H^2^ + 21.570 × H	Greece, men and women (N = 462; 40.3 ± 15.2 years)	0.99	2.65 kg	Height in meters Sex code: male = 1, female = 0
	Sardinha et al. (2023)	ALSM	BIA 101 BIVA PRO, Akern s.r.l., Italy	DXA	LSM_left body_ (kg) = 9.016 + 0.399 × RILB –91.962 × RITE + 1.229 × sex LSM_right body_ (kg) = 0.461 + 0.273 × RIRB + 0.006 × RIRT LSM _lower body_ (kg) = 7.998 + 0.284 × RILwB –100.561 × RITE + 1.559 × sex LSM_upper body_ (kg) = 1.560 + 0.102 × RIUB –23.420 × RITE + 0.717 × sex LSM_left leg_ (kg) = 4.756 + 0.067 × RILL –54.597 × RITE + 0.901 × sex LSM_right leg_ (kg) = 3.724 + 0.071 × RIRL –46.197 × RITE + 0.733 × sex LSM_left arm_ (kg) = 0.676 + 0.026 × RILA –11.398 × RITE + 0.346 × sex LSM_right arm_ (kg) = 1.034 + 0.024 × RIRA –12.272 × RITE + 0.388 × sex LSM_trunk_ (kg) = -10.039 + 0.015 × RIT + 160.945 × RITE	Portugal, men (N = 49; 33.3 ± 12.2 years) and women (N = 51; 33.3 ± 12.2 years)	0.95 0.95 0.95 0.96 0.94 0.95 0.96 0.95 0.88	1.80 kg 1.90 kg 1.40 kg 0.60 kg 0.80 kg 0.70 kg 0.30 kg 0.30 kg 2.60 kg	RILB: left body resistance index, RITE: resistance index of the ratio between trunk to extremities, RIRB: right body resistance index, RIRT right trunk resistance index, RILwB: lower body resistance index, RIUB: upper body resistance index, RILL: left leg resistance index, RIRL: right leg resistance index, RILA: left arm resistance index, RIRA: right arm resistance index, RIT: mean trunk resistance index calculated as the mean of the right and left trunk resistance indexes, Sex code: male = 1, female = 0
	*Segmental*								
	Oshima et al. (2010)	SMM	HBF-354, Omron Healthcare Co. Ltd, Japan	MRI	SMM (kg) = 0.126 × H^2^/Z + 1.937 × BSA –0.062 × age –2.186 × sex –2.881	Japan, men (N = 71; 40.5 ± 13.7 years) and women (N = 92; 43.4 ± 12.9 years)	0.89	1.65 kg	BSA: body surface area Sex code: male = 1, female = 2
MF-BIA	*Segmental*								
	Xu et al. (2020)	ALSM, FFM, and AFFM	BCA II, TFHT, China	DXA	ALSM (kg) = 7.950 + 2.334 × sex –0.034 × age + 0.145 × Wt –125.351 × R_50_/H^2^ + 43.063 × R_250_/H^2^ FFM (kg) = –13.563 + 5.469 × sex + 0.233 × Wt + 0.176 × H + 0.652 × H^2^/R_50_ –0.073 × age AFFM (kg) = –10.413 + 2.441 × sex + 0.124 × Wt + 0.096 × H + 0.278 × H^2^/R_50_ –0.024 × age	China, men (N = 13,973; 18–96 years) and women (N = 16,527; 18–96 years)	0.85 0.93 0.89 0.89	1.68 kg 2.33 kg 1.41 kg 2.03 kg	R50 and R250 represent whole body R measured at 50 and 250 kHz Sex code: male = 1, female = 0
BIS	*Foot to hand*								
	Hughes et al. (2015)	FFM	SFB7, ImpediMed Ltd, Australia	DXA	FFM (kg) = 0.432 × H^2^/R− 0.086 × age + 0.269 × Wt − 6.422 × sex + 16.429	Australia, men (N = 41; mean age 50.5 years) and women (N = 55; mean age 50.5 years)	0.94	3.33 kg	Data measured at 50 kHz Sex code: male = 0, female = 1

*ALSM* appendicular lean soft mass, *AFFM* appendicular fat-free mass, *BIS* bioelectrical impedance spectroscopy, *DXA* dual-energy X-ray absorptiometry, *FFM* fat-free mass, *H* height (cm), *LSM* lean soft mass, *MF-BIA* multifrequency bioelectrical impedance analysis, *MRI* Magnetic Resonance Image, *R* resistance, *R2* coefficient of determination, *SEE* standard error of estimation, *SF-BIA* single-frequency bioelectrical impedance analysis, *TBW* total body water, *Wt* body mass, *WHR* waist to hip ratio, *Xc* reactance, *Z* impedance, *4C* 4 compartmental model

### Bioelectrical impedance-based equations for athletes

Table 3 reports the characteristics of the 19 predictive equations developed in 11 studies on athletes. Fifteen predictive equations from 10 studies were developed with a SF-BIA, while four predictive equations from one study were developed with MF-BIA using bioelectrical parameters measured at 50 kHz.

**Table 3:** Predictive equations (N = 19) from studies (N = 11) on athletes

	Author	Variable	Analyzer	Reference	Equation	Country and participants characteristics	R^2^	SEE	Note
SF-BIA	*Foot to hand*								
	Fornetti et al. (1999)	FFM	BIA 101A, RJL Systems Inc., USA	DXA	FFM (kg) = 0.272 × H + 0.461 × Wt − 0.036 × R + 0.101 × Xc -11.567	USA, collegiate athletes, women (N = 66; 20.4 ± 1.5 years)	0.96	1.20 kg	
	Yannakoulia et al. (2000)	FFM	BIA 101, RJL Systems Inc., USA	DXA	FFM (kg) = 0.247 × Wt + 0.214 × H^2^/R + 0.191 × H − 14.96	Greece, professional dancers, women (N = 42; 21 ± 2 years)	0.83	1.45 kg	
	Matias et al. (2015)	TBW ECW	BIA 101 ASE, Akern s.r.l., Italy	Dilution techniques	TBW (kg) = 0.286 + 0.195 × H^2^/R + 0.385 × Wt + 5.086 × Sex ECW (kg) = 1.579 + 0.055 × H^2^/R + 0.127 × Wt + 0.006 × H^2^/Xc + 0.932 × Sex	Portugal, mixed athletes, men (N = 92; 21.2 ± 4.7 years) and women (N = 47; 20.6 ± 5.1 years)	0.91 0.70	2.42 kg 1.33 kg	Sex code: male = 1, female = 0
	Langer et al. (2016)	FFM	Quantum II, RJL Systems Inc, USA	DXA	FFM (kg) = 0.508 × Wt + 39.234 × (H^2^/R)^Log10^ − 48.263	Brazil, army cadets, men (N = 264; 19.3 ± 1.2 years)	0.87	2.30 kg	
	Koury et al. (2018)	FFM	BIA 450, Byodinamics corporation, USA	DXA	FFM_males_ (kg) = − 6.340 + 0.795 × age + 2.071 × skeletal maturity + 0.744 × H^2^/R FFM_females_ (kg) = − 2.615 + 0.603 × age + 0.954 × menarche occurrence + 0.713 × H^2^/R	Brazil, mixed athletes, boys (N = 165; 13.3 ± 1.1 years) and girls (N = 153; 13.3 ± 1.1 years)	0.92 0.84	2.74 kg 2.26 kg	Skeletal maturity code: immature = 0, mature = 1 Menarche code: no-occurrence = 0, occurrence = 1
	Sardinha et al. (2020)	ALSM	BIA 101 ASE, Akern s.r.l., Italy	DXA	ALSM_arms_ (kg) = 0.940 × sex + 0.042 × Wt + 0.080 × H^2^/R + 0.024 × Xc − 3.927 ALSM_legs_ (kg) = 1.983 × sex + 0.154 × Wt + 0.127 × H^2^/R − 1.147	Portugal, mixed athletes, men (N = 112; 22.5 ± 4.4 years) and women (N = 65; 22.1 ± 4.5 years)	0.89 0.81	0.61 kg 1.93 kg	Sex code: male = 0, female = 1
	Matias et al. (2021)	FFM	BIA 101, Akern s.r.l., Italy	4C	FFM (kg) = − 2.261 + 0.327 × H^2^/R + 0.525 × Wt + 5.462 × Sex	Portugal, mixed athletes, men (N = 72; 23.2 ± 5.0 years) and women (N = 23; 22.1 ± 4.6 years)	0.95	2.64 kg	Sex code: male = 1, female = 0
	Matias et al. (2022)	FFM	BIA 101 BIVA PRO, Akern s.r.l., Italy	DXA	FFM (kg) = − 8.865 + 0.437 × Wt + 0.186 × Xc + 0.415 × H^2^/R	Portugal, futsal players, men (N = 66; 23.3 ± 5.4 years)	0.89	2.38 kg	
	Campa et al. (2023)	FFM LSM ALSM	BIA 101 BIVA PRO, Akern s.r.l., Italy	DXA	FFM = − 7.729 + (Wt × 0.686) + H^2^/(R × 0.227) + Xc × 0.086) + age × 0.058 LSM = − 8.929 + Wt × 0.635 + H^2^/(R × 0.244) + Xc × 0.093 + age × 0.048 ALSM = − 24.068 + Wt × 0.347 + H^2^/(R × 0.308) + Xc × 0.152	Italy, elite soccer players participating in the Italian first league, men (N = 73; 25.2 ± 5.2 years)	0.97 0.96 0.88	1.0 kg 0.9 kg 1.4 kg	Height in meters
	Mauro et al. (2023)	FM	BIA 101 BIVA PRO, Akern s.r.l., Italy	DXA	FM = e^logFM^ where logFM = 0.300007 × Wt + 0.006438 × R/H^2^ − 0.03035	Italy, padel players, men (N = 15; 26.7 ± 11.8 years)	0.95	N/A	e = 2.72
	*Segmental*								
	Chao et al. (2011)	FFM	BC-418, Tanita corporation, Japan	DXA	FFM_upper-limb_ (kg) = − 0.746 + 0.028 × H^2^/Z _upper limb_ − 0.003 × age + 0.017 × Wt FFM_lower-limb_ (kg) = − 0.044 − 0.005 × H^2^/Z_lower limb_ − 0.054 × age + 0.203 × Wt FFM _whole body_ (kg) = − 1.146 + 0.212 × H^2^/Z _whole body_ − 0.187 × age + 0.780 × Wt FFM_trunk_ (kg) = FFM_whole body_ − 2 × FFM_lower limb_ − 2 × FFM_upper limb_	Taiwan, elite football players, men (N = 26; 20.6 ± 1.8 years)	0.74 0.78 0.95 0.89	0.31 kg 0.82 kg 1.71 kg 1.19 kg	Data measured at 50 kHz

*ALSM* appendicular lean soft mass, *BIS* bioelectrical impedance spectroscopy, *DXA* dual-energy X-ray absorptiometry, *ECW* extracellular water, *FFM* fat-free mass, *H* height (cm), *MF-BIA* multifrequency bioelectrical impedance analysis, *R* resistance, *R2* coefficient of determination, *SEE* standard error of estimation, *SF-BIA* single-frequency bioelectrical impedance analysis, *TBW* total body water, *Wt* body mass, *Xc* reactance, *Z *impedance, *4C* 4 compartmental model

### Bioelectrical impedance-based equations for elderly

Table 4 reports the characteristics of the 26 predictive equations developed in 17 studies on elderly subjects. Seventeen predictive equations from 11 studies were developed with a SF-BIA, while eight predictive equations from seven studies were developed with MF-BIA using bioelectrical parameters measured at 2, 50, and 250 kHz. One study provided a predictive equations using bioelectrical parameters measured with BIS using at a frequency of 5 kHz.

**Table 4:** Predictive equations (N = 26) from studies (N = 17) on elderly people

	Author	Variable	Analyzer	Reference	Equation	Country and participants characteristics	**R**^**2**^	**SEE**	**Note**
SF-BIA	*Foot to hand*								
	Svendsen et al. (1991)	FM	BIA 103, RJL Systems Inc., USA	DXA	FM_males_ (kg) = 20.85 – 1.75 × ST – 0.51 × WC + 44.72 × WHR – 0.05 × R – 0.63 × H^2^/R + 0.88 × Wt FM_females_ (kg) = − 80.92 – 0.20 × WC + 0.39 × SS + 0.48 × H – 0.54 × H^2^/R + 2.33 × BMI	Danmark, men (N = 23; 75 years) and women (N = 23; 75 years)	0.98 0.97	0.95 kg 1.81 kg	Height in meters
	Williams et al. (1995)	FFM	Mod. 1990B, Valhalla Scientific, USA	4C	FFM_males_ (kg) = 0.54 × H^2^/R + 0.13 × Wt + 0.13 × R − 0.11 × age + 8.7 FFM_females_ (kg) = 0.37 × H^2^/R + 0.16 × Wt + 11.94	USA, white men (N = 25; 68.5 ± 7.1 years) and women (N = 23; 65.0 ± 9.4 years)	0.87 0.96	1.50 kg 1.50 kg	Height in meters
	Roubenoff et al. (1997)	FFM	BIA 101, RJL Systems Inc., USA BCA analyzer, BCA, Inc., USA	DXA	FFM_male_ (kg) = 9.1536 + 0.4273 × H^2^/R + 0. 1926 × Wt + 0.0667 × Xc FFM_female_ (kg) = 7.7435 + 0.4542 × H^2^/R + 0. 1190 × Wt + 0.0455 × Xc	USA, White men (N = 116; 76.2 ± 5.6 years) and women (N = 167; 75.8 ± 6.6 years)	0.72 0.77	3.43 kg 2.09 kg	
	Dittmar et al. (2001)	BCM	BIA 2000-M, Data Input, Germany	TBK	BCM (kg) = 1.898 × H^2^/Xc − 0.051 × Wt + 4.180 × sex + 15.496	Germany, men (N = 55; 68.6 ± 5.4 years) and women (N = 55; 68.9 ± 5.7 y)	0.84	1.71 kg	Height in meters Sex code: male = 1, female = 0
	Haapala et al. (2002)	FFM	BIA 101, RJL Systems Inc., USA	DXA	FFM (kg) = − 128.06 × 1.85 × BMI − 0.63 × Wt + 1.07 × H – 0.03 × R + 10.0 × WHR	Finland, women (N = 93; 66.8 ± 3.2 years)	0.83	1.60 kg	
	Dey et al. (2003)	FFM	BIA 101, RJL Systems Inc., USA	4C	FFM (kg) = 11.78 + 0.499 × H^2^/R + 0.134 × Wt + 3.449 × sex	Sweden, men (N = 201; 70 years and N = 143; 75 years) and women (N = 299; 70 years and N = 180; 75 years)	0.95	2.64 kg	Sex code: male = 1, female = 0
	Rangel Peniche et al. (2015)	ALSM	Quantum X, RJL Systems Inc., USA	DXA	ALSM (kg) = − 0.05376 + 0.2394 × H^2^/R + 2.708 × sex + 0.065 × Wt	Mexico, men (N = 28; 69.3 ± 6.5 years) and women (N = 79; 69.3 ± 6.5 years)	0.91	1.01 kg	Sex code: male = 1, female = 0
	Sergi et al. (2015)	ALSM	BIA 101 ASE, Akern s.r.l., Italy	DXA	ALSM (kg) = − 3.964 + 0.227 × H^2^/R + 0.095 × Wt + 1.384 × sex + 0.064 × Xc	Italy, men (N = 117; 71.4 ± 5.4 years) and women (N = 179; 71.4 ± 5.4 years)	0.92	1.14 kg	Sex code: male = 1, female = 0
	De Rui et al. (2016)	FFM	BIA 101 ASE, Akern s.r.l., Italy	DXA	FFM_dominant arm_ (kg) = − 0.081 + 0.061 × H^2^/R + 0.010 × Wt + 0.299 × sex FFM_non dominant arm_ (kg) = − 0.026 + 0.014 × H^2^/R + 0.009 × Wt + 0.352 × sex FFM_dominant leg_ (kg) = − 0.462 + 0.027 × H^2^/R + 0.047 × Wt + 0.639 × sex + 0.026 × Xc FFM_non dominant leg_ (kg) = − 0.522 + 0.029 × H^2^/R + 0.045 × Wt + 0.569 × sex + 0.025 × Xc	Italy, Caucasian men (N = 117; 58 – 85 years) and women (N = 179; 58 – 85 years)	0.86 0.88 0.88 0.88		Sex code: male = 1, female = 0 R and Xc are referred region al measures
	Barbosa-Silva et al. (2019)	ALSM	Quantum II, RJL Systems Inc, USA	DXA	ALSM (kg) = 2.08 × sex + 0.04 × Wt + 0.24 × H^2^/R + 0.07 × Xc − 0.16	Brazil, men (N = 70; age > 60 years) and women (N = 111; female (age > 60 years)	0.89	1.34 kg	Sex code: male = 1, female = 0
	Toselli et al. (2021)	ALSM	BIA Analyzer Nutritional Solutions, USA	DXA	ALSM (kg) = 5.982 + 0.188 × H^2^/R + 0.014 × WC + 0.046 × Wt + 3.881 × sex − 0.053 × age	Brazil, Caucasian men (N = 26; 75.6 ± 7.8 years) and women (N = 92; 70.9 ± 6.6 years)	0.86	1.35 kg	Sex code: male = 1, female = 0
MF-BIA	*Foot to hand*								
	Barbosa-Silva et al. (2019)	ALSM	QuadScan 4000, Bodystat® UK	DXA	ALSM (kg) = 1.85 × sex + 0.03 × Wt + 0.31 × H^2^/R_50_ + 0.04 × Xc_50_ + 0.01 × Z_5_ − 8.16	Brazil, men (N = 70; age > 60 years) and women (N = 111; female (age > 60 years)	0.90	1.29 kg	R_50_: resistance at 50 kHz Xc_50_: reactance at 50 kHz Z_5_: impedance at 5 kHz Sex code: male = 1, female = 0
	Kim et al. 2014	ALSM	InBody 3.0, InBody Co., South Korea	DXA	ALSM (kg) = H^2^/R_250_ × 0.104 − age × 0.050 + sex × 2.954 + Wt × 0.055 + 5.663	Korea, men (N = 285; 71.1 ± 3.7 years) and women (N = 435; 71.0 ± 3.5 years)	0.89	1.35 kg	R_250_: resistance at 250 kHz Sex code: male = 1, female = 0
	Vermerien et al. (2018)	ALSM	QuadScan 4000, Bodystat® UK	DXA	ALSM (kg) = 0827 + 0.19 × H^2^/R + 2101 × sex + 0.079 × Wt	Belgium, men (N = 91; 83.3 ± 2.9 years) and women (N = 83; 83.3 ± 3.0 years)	0.89	1.45 kg	Data measured at 50 kHz Sex code: male = 1, female = 0
	Jeon et al. (2020)	ALSM	InBody S10, InBody Co., South Korea	DXA	ALSM (kg) = 0.276 × H^2^/R_250_ + 1.151 × sex + 0.059 × Xc_5_ + 0.429	Korea, men (N = 63; 76.4 ± 4.2 years) and women (N = 70; 76.1 ± 4.1 years)	0.93	1.01 kg	R_250_: resistance at 250 kHz, Xc_50_: reactance at 50 kHz Sex code: male = 1, female = 0
	Kim et al. (2022)	ALSM	BWA 2.0, InBody Co., South Korea	DXA	ALSM (kg) = 0.247 × H^2^/R_2_ + 1.254 × sex + 0.067 × Xc_5_ + 1.739	Korea, men (N = 63; 77.5 ± 4.1 years) and women (N = 68; 76.9 ± 4.4 years)	0.93	0.97 kg	R_2:_ resistance at 2 kHz, Xc_5_: reactance at 5 kHz Sex code: male = 1, female = 0
	*Segmental*								
	Yoshida et al. (2014)	ALSM	MC-980ª, Tanita Corporation, Japan	DXA	ALSM_males_ (kg) = 0.197 × H^2^/R + 0.179 × Wt − 0.019 ALSM_females_ (kg) = 0.221 × H^2^/R + 0.117 × Wt + 0.881	Japan, men (N = 141; age 73.7 ± 5.7 years) and women (N = 109; 73.2 ± 5.5 years)	0.87 0.89	0.98 kg 0.81 kg	Data measured at 50 kHz
	Jeon et al. (2020)	ALSM	InBody 770, InBody Co., South Korea	DXA	ALSM (kg) = 0.286 × H^2^/R_250_ + 1.367 × sex + 0.054 × Xc_50_ + 0.031 × Wt − 1.864	Korea, men (N = 63; 76.4 ± 4.2 years) and women (N = 70; 76.1 ± 4.1 years)	0.93	1.02 kg	R_250_: resistance at 250 kHz, Xc_50_: reactance at 50 kHz Sex code: male = 1, female = 0
BIS	*Foot to hand*								
	Tengvall et al. (2009)	SMM	Hydra 4200, Xitron Technologies, USA	DXA	SMM (kg) = − 24.021 + 0.33 × H − 0.031 × R + 0.083 × Xc_5_ − 1.58 × sex + 0.046 × Wt	Sweden, men (N = 48; mean age 75 years) and women (N = 50; mean age 75 years)	0.93	1.59 kg	Xc_5:_ reactance at 5 kHz Sex code: male = 0, female = 1

*ALSM* appendicular lean soft mass, *BCM* body cell mass, *BIS* bioelectrical impedance spectroscopy, *BMI* body mass index, *DXA* dual-energy X-ray absorptiometry, *FFM* fat-free mass, *H* height (cm), *MF-BIA* multifrequency bioelectrical impedance analysis, *R* resistance, *R*^*2*^ coefficient of determination, *SEE* standard error of estimation, SF-BIA single-frequency bioelectrical impedance analysis, *SMM* skeletal muscle mass, *SS* subscapular skinfold thickness, *ST* scapular to triceps skinfold thickness, *TBK* total body potassium, *TBW* total body water, *Wt* body mass, *WC* waist circumference, *WHR* waist to hip ratio, *Xc* reactance, *Z* impedance, *4C* 4 compartmental model

### Bioelectrical impedance-based equations for people with diseases

Table 5 reports the characteristics of the 16 predictive equations developed in 8 studies on subjects with diseases. Three studies presented seven predictive equations using SF-BIA, while six predictive equations from two studies were developed with MF-BIA using bioelectrical parameters measured at 50 and 100 kHz. Three studies developed three predictive equation using BIS at a frequency of 50 kHz.

**Table 5:** Predictive equations (N = 16) from studies (N = 8) on people with diseases

	Author	Variable	Analyzer	Reference	Equation	Country and participants characteristics	R^2^	SEE	Note
SF-BIA	*Foot to hand*								
	Kanellakis et al. (2010)	FFM	BIA 101, Akern s.r.l., Italy	DXA	FFM (kg) = 38.475 + 0.207 × Wt − 0.092 × R/H^2^ + 0.291 × Xc/H^2^	Greece, overweight and obese postmenopausal women (N = 131; 62.3 ± 6.0 years)	0.80	1.62 kg	Height in meters
	Scafoglieri et al. (2016)	AFM AFFM	BIA 101, Akern s.r.l., Italy	DXA	AFFM_Hologic_ (kg) = 4.957 + 0.196 × H^2^/R + 0.060 × Wt − 2.554 × sex AFFM_Lunar_ (kg) = 1.821 + 0.168 × H^2^/R + 0.132 × Wt + 0.017 × Xc − 1.931 × sex AFM_Hologic_ (kg) = − 4.716 − 0.142 × H^2^/R + 0.316 × Wt + 4.453 × sex − 0.040 × Xc AFM_Lunar_ (kg) = − 6.553 − 0.093 × H^2^/R + 0.272 × Wt + 4.295 × sex	Europe, functionally limited men (N = 88; 77.6 ± 6.9 years) and women (N = 203; 77.6 ± 6.9 years)	0.90 0.86 0.73 0.70	1.28 kg 1.37 kg 1.54 kg 1.53 kg	Sex code: male = 0, female = 1
	*Segmental*								
	Jiménez et al. (2012)	FFM FM	BC-418, Tanita Corporation, Japan	DXA	FFM_whole body_ (kg) = 18.240 − 4.395 × sex + 0.137 × Wt + 5,865.274 × H^2^ /Z FM_trunk_ (kg) = − 43.710 + 0.268 × HC + 0.207 × WC + 0.235 × Wt − 2232.18 × H^2^/Z	Spain, morbidly obese men (N = 35; 43.5 ± 11.8 years) and women (N = 124; 43.5 ± 11.8 years)	0.894 0.66	0.18 kg 9.10 kg	All circumferences are in cm Sex code: male = 1, female = 2
MF-BIA	*Foot to hand*								
	Scalfi et al. (1997)	TBW	Human IM Scan, Dietosystem, Italy	Dilution techniques	TBW (L) = 0.563 + H^2^/Z_100_ + 2.695	Italy, anorexic women (N = 19; 22.1 ± 4.9 years)	0.88	2.40 l	Z_100_: impedance at 100 kHz
	Choi et al. (2021)	FFM	InBody S10 InBody Co., South Korea	DXA	LA _FFM_ (kg) = − 3.759 + 0.204 × ZI_LA_ + 0.410 × Xc_LA_ + 0.019 × H − 0.007 × age RA_FFM_ (kg) = − 1.370 + 0.212 × ZI_RA_ + 0.054 × Xc_RA_ LL _FFM_ (kg) = − 4.089 + 0.162 × Xc_LL_ + 0.143 × ZI_LL_ + 0.039 × Wt + 0.006 × R_LL_ RL_FFM_ = − 3.715 + 0.009 × R_RL_ + 0.152 × ZI_RL_ + 0.139 × XcRL + 0.031 × Wt TR _FFM_ = − 2.061 + 0.046 × ZI_TR_ + 0.073 × Wt + 0.212 × H − 0.419 × R_TR_ + 0.041 × age	Korea, amputee men (N = 75; 43.6 ± 12 years)	0.90 0.73 0.90 0.93 0.76	0.29 kg 0.40 kg 0.91 kg 0.74 kg 1.51 kg	Data measured at 50 kHz ZI: impedance index; ZBPL: impedance; LA: left arm; RA: right arm; TR: trunk; LL: left leg; RL: right leg
BIS	*Foot to hand*								
	Van Baar et al. (2005)	ALSM	Hydra 4200, Xitron Technologies, USA	DXA	ALSM (kg) = − 6.296 + H^2^/R × 0.227 + Xc × 0.072 + sex × 9.909 + Wt × 0.072 + sex × age × − 0.098 + age × 0.054	Europe, frail men (N = 45; 80.4 ± 8.2 years) and women (N = 61; 77.5 ± 7.8 years)	0.92	1.19 kg	Data measured at 50 kHz Sex code: male = 1, female = 0
	Macdonald et al. (2006)	ALSM	Hydra 4200, Xitron Technologies, USA	DXA	ALSM (kg) = − 11.626 + 0.292 × H^2^/R + 0.06983 × Xc + 0.08553 × H − 2.092 × sex − 0.05 × age	England, nondiabetic CKD pre-dialysis (N = 75; 65.1 ± 12 years)	0.92	1.77 kg	Data measured at 50 kHz Sex code: male = 0, female = 1
	Lin et al. (2021)	ALSM	Body Composition Monitor, Fresenius Medical Care, Germany	DXA	ALSM (kg) = − 1.838 + 0.395 × TBW + 0.105 × W + 1.231 × sex − 0.026 × age	Taiwan, hemodialysis men (N = 115; 57.9 ± 11.3 years) and women (N = 99; 60.0 ± 12.5 years)	0.91	1.35 kg	Data measured at 50 kHz Sex code: male = 1 female = 0 TBW is estimated from bioelectrical data

*AFM* appendicular fat mass, *AFFM* appendicular fat-free mass, *ALSM* appendicular lean soft mass, *BCM* body cell mass, *BIS* bioelectrical impedance spectroscopy, *CKD* chronic kidney disease, *DXA* dual-energy X-ray absorptiometry, *ECW* extracellular water, *FFM* fat-free mass, *H* height (cm), *HC* hip circumference, *MF-BIA* multifrequency bioelectrical impedance analysis, *R* resistance, *R2* coefficient of determination, *SEE* standard error of estimation, *SF-BIA* single-frequency bioelectrical impedance analysis, *TBW* total body water, *Wt* body mass, *Xc* reactance, *Z* impedance

## Discussion

The present systematic review was designed to compile a list of BIA-based predictive equations for estimating body mass components in target populations. Specifically, we included only articles that developed new predictive models and excluded experimental designs that validated existing equations, mixed different populations, or used the two-component model as a reference for developing predictive equations. This resulted in 106 predictive equations that met high-standard procedures, organized based on the four BIA technologies (i.e., hand-to-hand, leg-to-leg, foot-to-hand, and segmental), the sampling frequency (i.e., single and multi-frequency) and five population categories (i.e., under 18 years old, adults, athletes, elderly, and people with diseases). Additionally, the geographical areas of the participants were identified. The present findings provide an updated starting point for researchers to identify potential gaps in the literature and develop further predictive equations. Moreover, this updated list facilitates the easy identification of the most accurate equations for specific populations and technologies, aiding practitioners in implementing best practices.

### Bioelectrical impedance-based equations for under 18 years old people

Nineteen predictive equations developed in 14 studies on subjects under 18 years old from general populations were included. Two of these studies were conducted in the USA, where no female participants were involved [55, 76], while the remaining 12 studies were performed in different countries around the world with both sexes included [37, 43–45, 57–59, 72–75, 100]. Out of the 19 equations, 12 were developed using foot to hand technology [37, 55, 58, 59, 72, 73, 75, 76, 100] and seven equations were developed using segmental technology [43–45, 57, 74]. Concerning foot-to-hand technology, four equations are suitable only for females [55, 76], while eight are suitable for both sexes [37, 58, 59, 72, 73, 75, 100]. Regarding segmental technology, five are suitable for both sexes [43–45, 57, 74]. Concerning foot-to-hand technology, four equations were found for estimating FFM in females only [55, 76] and two for both sexes [59, 73]. As for TBW, five equations are suitable for both sexes [37, 58, 72, 75, 100]. Considering segmental technology, five equations are available for estimating FFM in both sexes [43–45, 57, 74]. All 19 equations were developed at a single frequency of 50 kHz, although four predictive equations were developed with multifrequency devices but at the frequency of 50 kHz [43, 44, 57].

### Bioelectrical impedance-based equations for adults

Twenty-six predictive equations developed in 14 studies on adults were reviewed. Although most of the studies (n = 4) were conducted in the USA [34, 42, 54, 82], the largest participant number occurred in one Chinese study [81]. The remaining studies involved European [50, 52, 71, 77, 101], Eastern Asian [47, 78], and Australian [66] populations with a balanced number of male and female participants. Out of the 26 available equations, 22 were developed with foot to hand technology [34, 42, 47, 50, 52, 54, 61, 66, 71, 77, 78, 102], and four with segmental technology Concerning foot to hand technology, 17 equations are available for both sexes [42, 47, 52, 54, 61, 66, 71, 77, 102], while one is suitable for males [78] and two are suitable for females only [34, 50]. As for segmental technology, four equations are suitable for both sexes [80, 81]. Regarding foot-to-hand technology, five equations are suitable for estimating FFM in males [47, 52, 66, 71, 78] and six in females [34, 47, 50, 52, 66, 71]. Two equations are suitable for estimating TBW [42, 54], ten for estimating LSM [61, 77], and one for estimating SMM [82] in both sexes. Particularly, the equations predicting LSM were developed for the appendicular [61, 77] and trunk [61] body segments. Considering segmental technology, two equations for estimating FFM [81], one equation for estimating SMM [80], and one equation for estimating LSM [81] are available for males and females. Out of the equations for predicting FFM or LSM, both estimate the appendicular mass only [81]. All these equations were developed at a single frequency of 50 kHz, with the exception of four equations [66, 81] developed with a multifrequency devices that used bioelectrical parameters measured only at 50 kHz [66], as well as mixing frequencies sampled at 50 and 250 kHz [81].

### Bioelectrical impedance-based equations for athletes

Nineteen predictive equations developed in 11 studies on athletes were included. Four studies involved Portuguese male and female participants from different sport disciplines [39, 69, 84, 87], while two studies recruited male Brazilian army cadets [85] considered as very active individuals [103] and adolescents from various sport disciplines [104]. One study was conducted on USA collegiate female athletes from different sports [62], one on Greek elite female dancers [51], and two studies on elite male soccer players in Taiwan [88] and Italy [31]. One study con considered Italian paddle players [48]. Out of the 19 available equations, 15 were developed with foot to hand technology [31, 39, 48, 51, 62, 69, 84, 85, 87, 104], while four equations were developed using segmental technology [88]. Considering the foot to hand technology, 12 equations are suitable for male [31, 39, 48, 69, 84, 85, 87, 104] and eight for female people only [39, 51, 62, 69, 87, 104]. As for segmental technology, four equations are specific for males [88], and no equation is available for females. Regarding foot to hand technology, we found an equation for estimating FM in males [48], five equations for estimating FFM in males [31, 69, 84, 85, 104], and four in females only [51, 62, 69, 104]. Moreover, we retrieved one equation for estimating TBW [39], one for estimating ECW [39] and five for estimating LSM [31, 87], where three of them are suitable for both sexes [39, 87], and two in male only [31]. As for segmental technology, four equations for estimating whole-body, upper-limb, lower-limb, and trunk FFM are available for males only [88]. All these equations were developed at a single frequency of 50 kHz, even those developed using a multifrequency device [88].

### Bioelectrical impedance-based equations for elderly

Twenty-six predictive equations developed in 17 studies on the elderly were included. The selected studies encompassed Central[36, 38, 49] and Southern American [46, 67], European [53, 63, 89–91, 93, 95, 97], and Asian [68, 94] male and female participants. Out of the 26 available equations, 23 were developed with foot to hand technology [36, 38, 46, 49, 53, 63, 67, 89–93, 95, 97], while three equations were developed using segmental technology [68, 94]. Concerning foot-to-hand technology, three predictive equations were suitable for males [36, 49, 53] and four for females only [36, 49, 53, 97], while thirteen equations suitable for both sexes [38, 46, 63, 67, 89–93, 95]. Two different equations are available to determine FM in males and females [53], while there are two equations for assessing FFM in males [36, 49], three in females only [36, 49, 97], and five suitable for both sexes [89, 91]. There is one equation for estimating BCM [63], four equations for assessing appendicular LSM [46, 67, 92, 93], and one equation for assessing SMM [95] suitable for males and females. As for the segmental technology, only the appendicular LSM is currently possible to be determined using two equations suitable for both sexes [68, 94]. Sixteen equations were developed using single frequency devices at 50 kHz [36, 38, 46, 49, 53, 63, 67, 89–91, 97], while the equations developed with multifrequency devices were obtained measuring the bioelectrical parameters at 5 kHz [95], 50 kHz [93, 94], 250 kHz [92], or 50 and 250 kHz [68].

### Bioelectrical impedance-based equations for people with diseases

Sixteen predictive equations developed in eight studies on people with diseases were included. The selected studies included European [35, 40, 56, 64, 65, 98] and Asian [41, 99] male and female participants. Out of the 16 available equations, 14 were developed with foot to hand technology [40, 64, 98] and eight using segmental technology [40, 41, 56, 64, 65, 98, 99]. Regarding the foot to hand technology, there is one equation suitable for assessing FFM in overweight and obese postmenopausal females [98], four for appendicular FM and FFM in functionally limited male and female individuals [40], four for assessing FFM in amputee males [41], one for predicting TBW in anorexic females [56], and three for appendicular LSM in frail [64], chronic kidney [65], or hemodialysis male and female subjects [99]. Seven equations were developed using single frequency devices at 50 kHz [35, 40, 98], while 9 equations were made with multifrequency devices involving measures obtained at 50 [41, 64, 65, 99] and 100 kHz [56]. However, the use of BIA in individuals with underlying medical conditions necessitates a thorough consideration of the reliability features inherent in the measurements. This is imperative due to the heightened likelihood of fluctuations in water content, which may manifest more frequently in this cohort compared to other populations [105].

### Limitations of the review and future perspectives

**Fig. 7:**
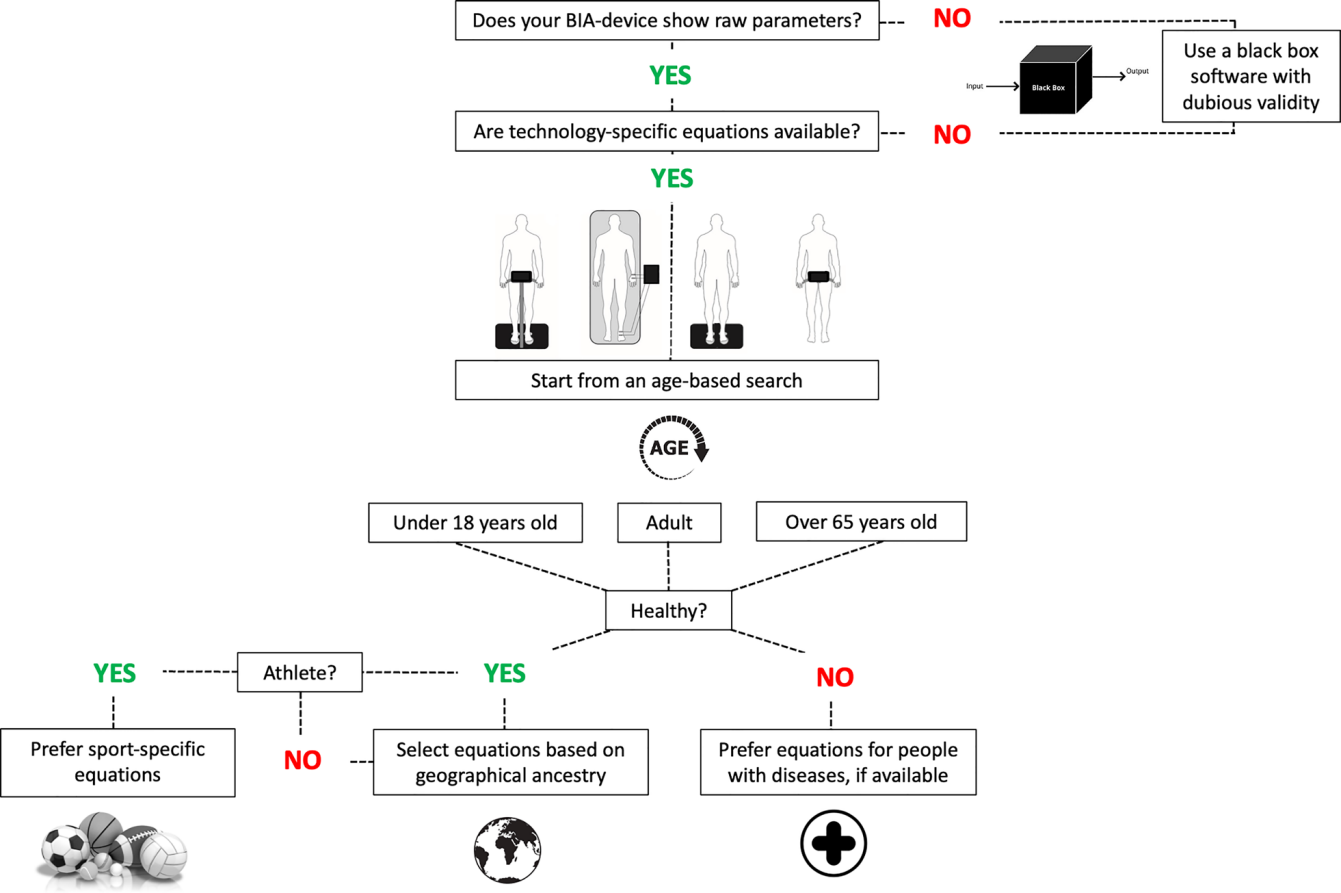
Procedures for accurately selecting predictive equations for assessing body composition using the bioelectrical impedance analysis

One limitation of the current manuscript may concern the grouping of predictive equations based on the BIA technology, since different devices using the same technology may yield different outcomes due to potential between-device inconsistencies [106], even though agreement has been observed [21]. Therefore, new comparisons between different devices of the same technology should be conducted. Other considerations could be made, not merely as limitations of the present manuscript but intrinsic to BIA. First, considering the lack of agreement between bioelectrical values obtained at different frequencies [21, 107], the sampling frequency information has been reported for each of the included studies. In this regard, future studies should clarify the advantages of using multi-frequency devices given that most predictive equations predominantly include measures obtained at a single frequency of 50 kHz. Second, the procedures for BIA were updated in 2004 [15] and further in 2020 for athletes [2]. Therefore, inconsistencies in methodological procedures (e.g., the standardization of food and beverage intake before the assessment, electrode placement, skin cleansing) may have impacted the final outcomes for each study [108]. Third, the standard error is often reported as absolute values of body mass, and this does not take into account how much each error is related to the total body mass. For example, a standard error of 1.5 kg in a female population with a mean FFM equal to 50 kg is less precise than in men with a mean FFM equal to 80 kg. Fourth, even though some variables are not directly estimated by the BIA (e.g., FM and ICW), these can be easily derived as the difference between the body mass and the FFM (for FM) or the total water and the ECW (for ICW). Lastly, the utilization of multicomponent models to estimate body composition may be susceptible to error propagation, stemming from the use of multiple methods for reference body composition values. A procedure for identifying the most valid equation for predicting body composition is illustrated in Fig. 7. There is reason to believe that such procedures will be considered in the development of AI-based software systems in the future.

## Conclusions

Numerous predictive equations for quantifying body composition with BIA have been developed using high-standard procedures. However, upon categorizing these equations based on BIA technology, target population, and estimable body mass components, the overall availability of predictive equations seems limited. Notably, no predictive equations for hand to hand and leg to leg technology are included here, implying that these BIA devices predominantly employ procedures of low quality, often unknown, and lacking scrutiny, with equations typically owned by manufacturers. In contrast, there are several predictive equations available for foot to hand and segmental technology. However, the accuracy of estimating certain body components is compromised due to the absence of population-specific predictive equations. Given the necessity for reference values and BIA-based predictive equations to be population- and technology-specific, the current findings underscore the urgency of developing new predictive equations tailored to specific BIA technology, population characteristics, and body mass components. Nevertheless, the foot to hand technology stands out with the highest number of predictive equations, offering the most accurate estimation of body composition. That said, the predictive equations available so far do not cover all possible combinations of technology, population and body mass component. Hence, the present manuscript may be helpful to pinpoint what has been done so far and what is currently lacking, so to generate novel predictive equations in cases not covered by the present literature. This may help to increase the trust of the practitioners in the BIA, often doubtful about the goodness of the results provided by inaccurate equations included in the device. Practitioners now possess an updated list of predictive equations for assessing body composition.

### Supplementary Information

Additional file1 (DOCX 40 KB)

## Data Availability

No new data were created or analyzed in this study. Data sharing is not applicable to this article.
