# Supplementary material for: High-standard predictive equations for estimating body composition using bioelectrical impedance analysis: a systematic review
Source: J Transl Med. 2024 May 29;22:515. doi: 10.1186/s12967-024-05272-x (PMC11137940; doi:10.1186/s12967-024-05272-x)
Supplement: Supplementary file 1 — Additional file1 (DOCX 40 KB) [file 12967_2024_5272_MOESM1_ESM.docx]

**Supplementary table 1**. Quality of included studies (N=64) using Quadas-2 (Quality Assessment of Diagnostic Accuracy Studies)

|  | **Risk of Bias** | | | | **Applicability Concerns** | | |
| --- | --- | --- | --- | --- | --- | --- | --- |
| **Studies** | **Patient selection** | **Index test** | **Reference standard** | **Flow and timing** | **Patient selection** | **Index test** | **Reference standard** |
| 1. Lukaski et al. (1988) | 🙂 | 🙂 | 🙂 | 🙂 | ☹ | 🙂 | 🙂 |
| 1. Heitmann et al. (1990) | 🙂 | 🙂 | 🙂 | 🙂 | ☹ | 🙂 | 🙂 |
| 1. Svendsen et al. (1991) | 🙂 | 🙂 | 🙂 | 🙂 | ☹ | 🙂 | 🙂 |
| 1. Zillikens et al. (1991) | 🙂 | 🙂 | 🙂 | 🙂 | ☹ | 🙂 | 🙂 |
| 1. Guo et al. (1993) | 🙂 | 🙂 | 🙂 | 🙂 | ☹ | 🙂 | 🙂 |
| 1. Williams et al (1995) | 🙂 | 🙂 | 🙂 | 🙂 | ☹ | 🙂 | 🙂 |
| 1. Roubenoff et al. (1997) | 🙂 | 🙂 | 🙂 | ? | 🙂 | 🙂 | 🙂 |
| 1. Scalfi et al. (1997) | 🙂 | 🙂 | 🙂 | 🙂 | ☹ | 🙂 | 🙂 |
| 1. Jakicic et al. (1998) | 🙂 | 🙂 | 🙂 | ☹ | 🙂 | 🙂 | 🙂 |
| 1. Fornetti et al. (1999) | 🙂 | 🙂 | 🙂 | 🙂 | ? | 🙂 | 🙂 |
| 1. Janssen et al (2000) | 🙂 | 🙂 | 🙂 | 🙂 | 🙂 | 🙂 | 🙂 |
| 1. Yannakoulia et al. (2000) | 🙂 | 🙂 | 🙂 | 🙂 | ☹ | 🙂 | 🙂 |
| 1. Dittmar et al. (2001) | 🙂 | 🙂 | 🙂 | 🙂 | ? | 🙂 | 🙂 |
| 1. Morrison et al. (2001) | 🙂 | 🙂 | 🙂 | 🙂 | 🙂 | 🙂 | 🙂 |
| 1. Haapala et al. (2002) | 🙂 | 🙂 | 🙂 | 🙂 | 🙂 | 🙂 | 🙂 |
| 1. Dey et al. (2003) | 🙂 | 🙂 | 🙂 | 🙂 | 🙂 | 🙂 | 🙂 |
| 1. Kyle et al. (2003) | 🙂 | 🙂 | 🙂 | 🙂 | 🙂 | 🙂 | 🙂 |
| 1. Leman et al. (2003) | 🙂 | 🙂 | 🙂 | ? | ☹ | 🙂 | 🙂 |
| 1. Pietrobelli et al. (2003) | 🙂 | 🙂 | 🙂 | 🙂 | ☹ | 🙂 | 🙂 |
| 1. Masuda et al. (2004) | 🙂 | 🙂 | 🙂 | 🙂 | ☹ | 🙂 | 🙂 |
| 1. Kontogianni et al. (2005) | 🙂 | 🙂 | 🙂 | 🙂 | ? | 🙂 | 🙂 |
| 1. Van Baar et al. (2005) | 🙂 | 🙂 | 🙂 | 🙂 | ? | 🙂 | 🙂 |
| 1. Macdonald et al. (2006) | 🙂 | 🙂 | 🙂 | 🙂 | ? | 🙂 | 🙂 |
| 1. Rush et al. (2006) | 🙂 | 🙂 | 🙂 | 🙂 | ☹ | 🙂 | 🙂 |
| 1. Nielsen et al. (2007) | 🙂 | 🙂 | 🙂 | 🙂 | ☹ | 🙂 | 🙂 |
| 1. Wickramasinghe et al. (2007) | 🙂 | 🙂 | 🙂 | 🙂 | 🙂 | 🙂 | 🙂 |
| 1. Kanellakis et al. (2009) | 🙂 | 🙂 | 🙂 | 🙂 | 🙂 | 🙂 | 🙂 |
| 1. Tengvall et al. (2009) | 🙂 | 🙂 | 🙂 | 🙂 | 🙂 | 🙂 | 🙂 |
| 1. Oshima et al. (2010) | 🙂 | 🙂 | 🙂 | 🙂 | 🙂 | 🙂 | 🙂 |
| 1. Sluyter et al. (2010) | 🙂 | 🙂 | 🙂 | 🙂 | 🙂 | 🙂 | 🙂 |
| 1. Chao et al. (2011) | 🙂 | 🙂 | 🙂 | 🙂 | 🙂 | 🙂 | 🙂 |
| 1. Liu et al (2011) | 🙂 | 🙂 | 🙂 | 🙂 | 🙂 | 🙂 | 🙂 |
| 1. Jiménez et al. (2012) | 🙂 | 🙂 | 🙂 | ☹ | 🙂 | 🙂 | 🙂 |
| 1. Kim et al. (2014) | 🙂 | 🙂 | 🙂 | 🙂 | 🙂 | 🙂 | 🙂 |
| 1. Yoshida et al. (2014) | 🙂 | 🙂 | 🙂 | 🙂 | 🙂 | 🙂 | 🙂 |
| 1. Hughes et al. (2015) | 🙂 | 🙂 | 🙂 | 🙂 | ? | 🙂 | 🙂 |
| 1. Rangel Peniche et al. (2015) | 🙂 | 🙂 | 🙂 | ? | 🙂 | 🙂 | 🙂 |
| 1. Sergi et al. (2015) | 🙂 | 🙂 | 🙂 | 🙂 | 🙂 | 🙂 | 🙂 |
| 1. De Rui et al. (2016) | 🙂 | 🙂 | 🙂 | 🙂 | 🙂 | 🙂 | 🙂 |
| 1. Langer et al. (2016) | 🙂 | 🙂 | 🙂 | 🙂 | 🙂 | 🙂 | 🙂 |
| 1. Matias et al. (2016) | 🙂 | 🙂 | 🙂 | ? | 🙂 | 🙂 | 🙂 |
| 1. Scafoglieri et al. (2016) | 🙂 | 🙂 | 🙂 | ? | 🙂 | 🙂 | 🙂 |
| 1. Koury et al. (2018) | 🙂 | 🙂 | 🙂 | 🙂 | 🙂 | 🙂 | 🙂 |
| 1. Vermerien et al. (2018) | 🙂 | 🙂 | 🙂 | 🙂 | 🙂 | 🙂 | 🙂 |
| 1. Barbosa-Silva et al. (2019) | 🙂 | 🙂 | 🙂 | 🙂 | ? | 🙂 | 🙂 |
| 1. Dasgupta et al. (2019) | 🙂 | 🙂 | 🙂 | 🙂 | 🙂 | 🙂 | 🙂 |
| 1. Van Zyl et al. (2019) | 🙂 | 🙂 | 🙂 | 🙂 | ☹ | 🙂 | 🙂 |
| 1. Jeon et al. (2020) | 🙂 | 🙂 | 🙂 | 🙂 | ? | 🙂 | 🙂 |
| 1. Kanellakis et al. (2020) | 🙂 | 🙂 | 🙂 | 🙂 | 🙂 | 🙂 | 🙂 |
| 1. Nguyen et al. (2020) | 🙂 | 🙂 | 🙂 | 🙂 | ☹ | 🙂 | 🙂 |
| 1. Sardinha et al. (2020) | 🙂 | 🙂 | 🙂 | 🙂 | 🙂 | 🙂 | 🙂 |
| 1. Xu et al. (2020) | 🙂 | 🙂 | 🙂 | 🙂 | 🙂 | 🙂 | 🙂 |
| 1. Choi et al. (2021) | 🙂 | 🙂 | 🙂 | ? | ? | 🙂 | 🙂 |
| 1. Gutiérrez-Marin et al. (2021) | 🙂 | 🙂 | 🙂 | 🙂 | ☹ | 🙂 | 🙂 |
| 1. Kourkoumelis et al. (2021) | 🙂 | 🙂 | 🙂 | 🙂 | 🙂 | 🙂 | 🙂 |
| 1. Lin et al (2021) | 🙂 | 🙂 | 🙂 | 🙂 | 🙂 | 🙂 | 🙂 |
| 1. Matias et al. (2021) | 🙂 | 🙂 | 🙂 | 🙂 | ? | 🙂 | 🙂 |
| 1. Toselli et al. (2021) | 🙂 | 🙂 | 🙂 | 🙂 | ☹ | 🙂 | 🙂 |
| 1. Da Costa et al. (2022) | 🙂 | 🙂 | 🙂 | 🙂 | 🙂 | 🙂 | 🙂 |
| 1. Matias et al. (2022) | 🙂 | 🙂 | 🙂 | 🙂 | 🙂 | 🙂 | 🙂 |
| 1. Kim et al. (2022) | 🙂 | 🙂 | 🙂 | 🙂 | ? | 🙂 | 🙂 |
| 1. Campa et al. (2023) | 🙂 | 🙂 | 🙂 | 🙂 | 🙂 | 🙂 | 🙂 |
| 1. Mauro et al. (2023) | 🙂 | 🙂 | 🙂 | 🙂 | ☹ | 🙂 | 🙂 |
| 1. Sardinha et al. (2023) | 🙂 | 🙂 | 🙂 | 🙂 | ? | 🙂 | 🙂 |

🙂: low risk; ☹: high risk; ?: unclear risk
